# Using co‐designed films to reduce burden and improve mood among Indian family carers: Results from a pilot randomized clinical trial

**DOI:** 10.1002/alz70858_097769

**Published:** 2025-12-24

**Authors:** Bianca Brijnath, Rachita Rao, Upasana Baruah, Josefine Antoniades, Mathew Varghese, Claudia Cooper, Briony Dow, Mike Kent, Santosh Loganathan

**Affiliations:** ^1^ La Trobe University, Bundoora, VIC, Australia; ^2^ National Institute of Mental Health and Neuro Sciences, Bengaluru, India; ^3^ National Ageing Research Institute, Melbourne, VIC, Australia; ^4^ St John's Medical College, Bengaluru, Karnataka, India; ^5^ Queen Mary University of London, London, United Kingdom; ^6^ Curtin University, Bentley, Western Australia, Australia; ^7^ National Institute of Mental Health and Neurosciences, Bangalore, Karnataka, India

## Abstract

**Background:**

We coproduced 10 short films on dementia care with Indian family carers and clinicians. The aim of this study was to assess the feasibility and effect of these films on family carers.

**Methods:**

A single‐blind, parallel‐group pilot randomized clinical trial conducted with adults carers (18+), speaking English, Hindi and/or Kannada, and owning a smartphone. The trial was community‐based, conducted online, between 15 October 2023 and 31 May 2024, with follow‐up at 3 and 6 months. The primary feasibility outcome was >70% of participants expressing interest consenting to participate, and >80% participation in follow up. The primary effect outcome was reduction in caregiver burden, as measured by the Zarit Burden Interview. Secondary outcomes were improvement in caregiver mood and quality of life, as measured by the Center for Epidemiological Studies Depression 10‐item scale and the World Health Organization Quality of Life Scale (WHOQOL‐BREF) respectively.

**Results:**

At baseline, there were 50 participants (26 [52%] women); mean (SD) age was 43.48 (15.63 years); mean years of care was 6.24 (0.85 years); and 36 (72%) lived in an urban area. A total of 47 (94%) spoke English; 38 (76%) Hindi; and 21 (42%) Kanada. Preliminary analysis of the primary feasibility outcome showed 50 of 58 (86.21%) expressing interest in the study going onto participate, with 43 (86%) and 45 (90%) participating at 3 and 6 months. Interaction effect revealed non‐significant improvements between intervention and control at 6 months for the primary outcome – burden [Intervention = 38.14 (16.01); Control = 42.86 (19.32), *p* = 0.083] and the secondary outcome – mood [Intervention = 11.32 (6.75); Control = 12.35 (6.44), *p* = 0.169]. Similarly, there were no significant effects in the quality‐of‐life domains except for carer self‐rated physical health (Domain 1) at 6 months [Intervention = 64.93 (17.76); Control = 58.54 (22.426); *p* = 0.036].

**Conclusions:**

The trial design is feasible, the films only slightly reduce carer burden and improve mood but significantly improve carer self‐rated physical health. Given smartphone penetration in India (>830 million users), coproduced short online films may be a promising way to support families caring for a person with dementia at home.

